# Identification of the shared gene signatures and molecular mechanisms between multiple sclerosis and non-small cell lung cancer

**DOI:** 10.3389/fimmu.2023.1180449

**Published:** 2023-05-12

**Authors:** Jingyun Yang, Xiaofei Hu, Yu Wang, Wenying Liu, Mengjie Zhang, Anmei Zhang, Bing Ni

**Affiliations:** ^1^ Department of Oncology, Xinqiao Hospital, Army Medical University (Third Military Medical University), Chongqing, China; ^2^ Department of Pathophysiology, College of High Altitude Military Medicine, Army Medical University (Third Military Medical University), Chongqing, China; ^3^ Department of Nuclear Medicine, Southwest Hospital, Army Medical University (Third Military Medical University), Chongqing, China; ^4^ Medical Research Institute, Southwest University, Chongqing, China; ^5^ Department of Dermatology, Southwest Hospital, Army Medical University (Third Military Medical University), Chongqing, China

**Keywords:** shared gene signature, molecular mechanisms, multiple sclerosis, non-small cell lung cancer, shared local immune environment

## Abstract

**Introduction:**

The association between multiple sclerosis (MS) and non-small cell lung cancer (NSCLC) has been the subject of investigation in clinical cohorts, yet the molecular mechanisms underpinning this relationship remain incompletely understood. To address this, our study aimed to identify shared genetic signatures, shared local immune microenvironment, and molecular mechanisms between MS and NSCLC.

**Methods:**

We selected multiple Gene Expression Omnibus (GEO) datasets, including GSE19188, GSE214334, GSE199460, and GSE148071, to obtain gene expression levels and clinical information from patients or mice with MS and NSCLC. We employed Weighted Gene Co-expression Network Analysis (WGCNA) to investigate co-expression networks linked to MS and NSCLC and used single-cell RNA sequencing (scRNA-seq) analysis to explore the local immune microenvironment of MS and NSCLC and identify possible shared components.

**Results:**

Our analysis identified the most significant shared gene in MS and NSCLC, phosphodiesterase 4A (PDE4A), and we analyzed its expression in NSCLC patients and its impact on patient prognosis, as well as its molecular mechanism. Our results demonstrated that high expression of PDE4A was associated with poor prognoses in NSCLC patients, and Gene Set Enrichment Analysis (GSEA) revealed that PDE4A is involved in immune-related pathways and has a significant regulatory effect on human immune responses. We further observed that PDE4A was closely linked to the sensitivity of several chemotherapy drugs.

**Conclusion:**

Given the limitation of studies investigating the molecular mechanisms underlying the correlation between MS and NSCLC, our findings suggest that there are shared pathogenic processes and molecular mechanisms between these two diseases and that PDE4A represents a potential therapeutic target and immune-related biomarker for patients with both MS and NSCLC.

## Introduction

In recent years, the investigation of cancer risks in various chronic diseases has gained increasing attention in the scientific community. Notably, a long-term study conducted in Norway suggests that patients with multiple sclerosis (MS) may face an elevated risk of developing cancers, particularly respiratory, urogenital, and central nervous system cancers, with a 66% increased risk of respiratory system cancers (1). It is worth noting that cancer has been suggested as one of the principal causes of mortality among MS patients, who have a significantly reduced life expectancy (2). The etiology of MS involves a combination of autoimmunity, viral infection, genetic susceptibility, environmental factors, and individual predisposing factors (3). Notably, the malfunction and activation of self-reactive immune cells may underlie the pathogenesis of MS.

The association between MS and cancer risks has been extensively investigated, with evidence suggesting that MS patients may exhibit health behaviors associated with increased lung cancer risk, including smoking, lack of exercise, and obesity. Additionally, neurologic adverse events, including MS, have been identified as rare but potentially fatal complications of immunotherapy using immune checkpoint inhibitors (ICIs) for lung cancer, further highlighting the potential connection between MS and lung cancer (4). A few studies support an inflammatory patient-dependent immune-mediated component for MS, identifying genetic and environmental risk factors and showing that the pathogenic mechanism of T cell-mediated MS is similar to that of ICIs (5, 6), which suggests that MS and lung cancer might share common risk factors and potentially similar pathogenic mechanisms. Non-small cell lung cancer (NSCLC) accounts for 85% of all cases of lung cancer (7). However, to date, there have been few studies exploring the molecular mechanisms underlying the correlation between MS and NSCLC, and the common pathogenic mechanisms of MS and NSCLC are still unclear. As NSCLC has a high incidence in MS, investigating the mechanisms by which MS’s autoimmune processes promote NSCLC development can provide unique insights into the complex events behind NSCLC occurrence and help identify potential diagnostic and prognostic biomarkers and therapeutic targets.

In this study, we employed bioinformatics analysis techniques to uncover shared mechanisms and potential therapeutic targets for both diseases. The identification of these mechanisms and targets may aid in improving the management of MS patients and lead to earlier detection and treatment of NSCLC.

## Methods

### Data collection and processing

We searched MS data using the keyword “multiple sclerosis” and NSCLC data using the keyword “non-small cell lung cancer” within the Gene Expression Omnibus (GEO) dataset collection. The following conditions were required for each dataset: 1) the datasets must be complete and correct. 2) The datasets must be normalized using the corresponding method, such as the robust multi-array average (RMA) method. 3) The number of samples in each group must be more than three to ensure the accuracy of the conclusion. Following the above criteria, we finally obtained adequate datasets including GSE19188, GSE214334, GSE199460, and GSE148071, some of which included clinical information on patients, such as age and gender. GSE19188 contained 91 NSCLC and 65 adjacent normal lung tissue samples. GSE214334 contained three relapse–remitting MS, four primary progressive MS, four secondary progressive MS, and seven non-MS control white matter tissues. GSE199460 contained three experimental autoimmune encephalomyelitis (EAE) and three controls, which extracted brains from myelin oligodendrocyte glycoprotein (MOG)-induced EAE at the peak of the disease and control mice to isolate single cells following single-cell RNA-sequencing (scRNA-seq) using a microdroplet-based method from 10X Genomics. GSE148071 contained 42 scRNA-seq of tumor tissues of NSCLC patients. Moreover, to elevate the reliability of our conclusion, we also enrolled and filtered The Cancer Genome Atlas (TCGA) lung squamous cell carcinoma (LUSC) and lung adenocarcinoma (LUAD) RNA-seq data, as well as The Genotype-Tissue Expression (GTEx) project lung tissue RNA-seq data, and we excluded those data with incomplete clinical and survival information.

### Analysis of bulk gene expression data

We analyzed bulk gene expression data mainly using *RNAseqStat* (0.1.0) R package, which was an integrated tool for processing gene expression data. After reading the corresponding datasets and group information, the pipeline analysis began. First, principal component analysis (PCA) of all samples in each dataset was performed to pre-check sample distribution. Second, the correlation among samples and the standard deviation of genes were calculated for the evaluation of potential relationships among samples. Third, after quality control (QC), differentially expressed genes (DEGs) were computed and exhibited by volcano plot and heatmap. Fourth, we performed gene ontology (GO) and Kyoto Encyclopedia of Genes and Genomes (KEGG) enrichment of upregulated or downregulated genes in each dataset using DEGs. Moreover, further enrichment analysis was performed on Metascape. After that, a portion of crucial pathways or processes was further performed in Gene Set Enrichment Analysis (GSEA) and visualization. Finally, we used the Venn tool for the identification of the shared gene signatures of two diseases.

### Weighted gene co-expression network analysis

We used the Weighted Gene Co-Expression Network Analysis (WGCNA) tool to evaluate gene expression patterns in MS and NSCLC. The input data were the gene expression matrix and clinical information of each dataset. After loading the gene expression matrix, the missing values were checked, and the outliers were identified. Samples were clustered to figure out whether there were outliers. Then, the expression networks were constructed, and gene modules were identified; 7 were chosen for the soft power of MS and 5 for NSCLC in consideration of the best soft power estimation. Then, the modules were associated with phenotypic data and identified hub genes. Finally, the hub gene co-expression networks were constructed.

### Construction of protein–protein interaction network

We constructed a protein–protein interaction (PPI) network with the help of STRING (https://string-db.org/). After inputting sharing proteins of MS and NSCLC identified by the above process, we constructed the PPI network including many edges and nodes, representing a potential relationship between two proteins. We performed GeneMANIA (https://genemania.org) to help to predict the function of core shared genes of MS and NSCLC and to construct a PPI network.

### ScRNA-seq analysis process

ScRNA-seq analysis was performed on GSE199460 and GSE148071. The data went through the process of normalizing, finding variables, scaling, running PCA, scoring jack straw, finding neighbors, finding clusters, and running t-distributed stochastic neighbor embedding (tSNE) with the help of the *Seurat* package. After annotation of single cells according to the expression of markers, DEGs and cell fractions were calculated and visualized by the *scRNAtoolVis* package. The *CellChat* package was used to construct and visualize intercellular communication networks.

### Protein intensity verification

Protein intensity was the result of gene expression, while NSCLC patients had diverse protein patterns. The Human Protein Atlas (https://www.proteinatlas.org/) was an online web tool for evaluating protein patterns for cancer and normal tissues using immunohistochemistry (IHC). By carefully searching and filtering, the expression of the given protein in NSCLC tissue and normal lung tissue was finally obtained.

### Survival analysis

Survival analysis of NSCLC patients was performed on Kaplan–Meier Plotter (http://kmplot.com/analysis/). Patients were divided into two groups according to given gene expression. Immune infiltration condition was used for further stratified analysis to uncover the potential relationship between a given gene and the immune microenvironment of NSCLC patients.

### Immune infiltration analysis

We used the online immune infiltration analysis tool TIMER 2.0 (http://timer.cistrome.org/) for evaluating the special relationship between a given gene and the immune microenvironment of NSCLC. TIMER 2.0 is a comprehensive resource for the systematic analysis of immune infiltrates across diverse cancer types. The web server provided immune infiltrate abundances estimated by multiple immune deconvolution methods, and we used it to generate figures dynamically to explore tumor immunological, clinical, and genomic features comprehensively. A portion of immune infiltration analysis was facilitated and visualized with Aclbi (www.aclbi.com).

### Drug sensitivity analysis

Genomics of Drug Sensitivity in Cancer (GDSC) (https://www.cancerrxgene.org) was used to predict drug treatment response based on specific gene expression. A total of 1,017 NSCLC patients were taken into consideration. IC50 was an important indicator in evaluating drug efficacy and sample treatment response. This tool was based on GDSC, the largest open pharmacogenomics database at present.

## Results

### Evaluation of shared local immune environment between MS and NSCLC

**Figure 1:**
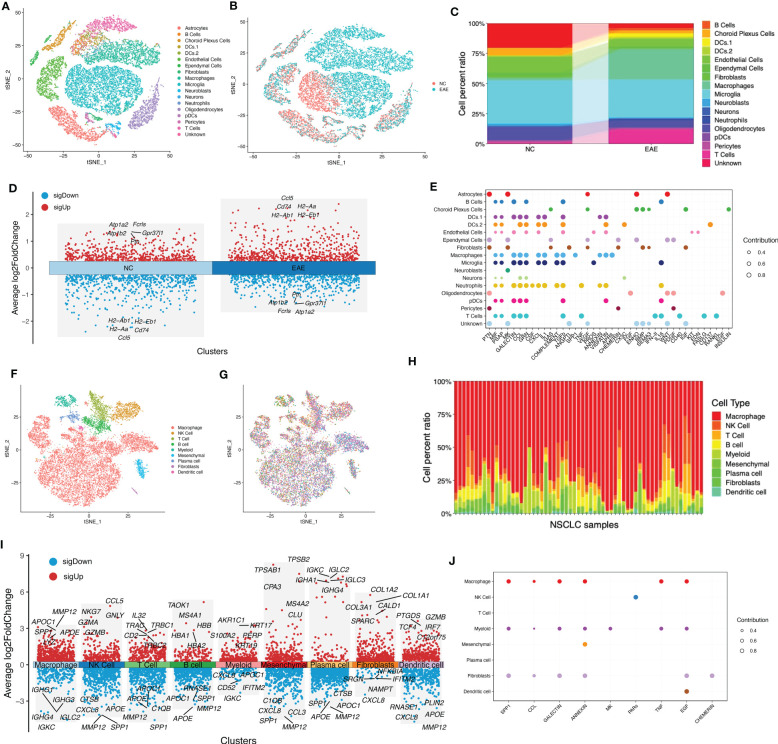
Evaluation of shared local immune environment between MS and NSCLC. **(A)** tSNE plot of scRNA-seq data of three EAE and three NC samples grouped by cell types. **(B)** tSNE plot of scRNA-seq data of three EAE and three NC samples grouped by sample types. **(C)** Cell fraction of different cell types in EAE and NC samples. **(D)** Volcano plot of upregulated and downregulated DEGs of EAE and NC groups. **(E)** Communication patterns of target cells in MS. **(F)** tSNE plot of scRNA-seq data of 42 NSCLC samples grouped by cell types. **(G)** tSNE plot of scRNA-seq data of 42 NSCLC samples grouped by samples. **(H)** Cell fraction of different cell types in NSCLC samples. **(I)** Volcano plot of upregulated and downregulated DEGs in each cell type. **(J)** Communication patterns of target cells in NSCLC. MS, multiple sclerosis; NSCLC, non-small cell lung cancer; tSNE, t-distributed stochastic neighbor embedding; scRNA-seq, single-cell RNA-sequencing; EAE, experimental autoimmune encephalomyelitis; NC, normal control; DEGs, differentially expressed genes.

In MS, an immune-related disease, the local immune environment was one of the most important factors affecting its progression. Considering the fact that NSCLC has a high incidence in MS, we speculated whether there was a shared local immune environment between NSCLC and MS. We used scRNA-seq data of the EAE mouse model to imitate the occurrence of MS. Our results showed that EAE had a unique local immune environment when compared with normal control (NC), including a reduced B-cell faction, increased macrophage fraction, reduced fibroblast fraction, and reduced neutrophils (**Figures 1A–C**). A portion of immune-related genes was differentially expressed in the EAE and NC groups, such as Cd74 and Ccl5 (**Figure 1D**). A portion of cells showed a potential relationship in secreted signaling (**Figure 1E**). As for NSCLC, different patients had diverse immune infiltration (**Figures 1F–H**). However, a portion of immune components in NSCLC was the same as that in MS, such as macrophage, B cell, and fibroblast. These immune cells had different gene expression patterns (**Figure 1I**). Similarly, these immune components showed a potential relationship in secreted signaling (**Figure 1J**).

### Uncovering gene characteristics during the progression of MS

**Figure 2:**
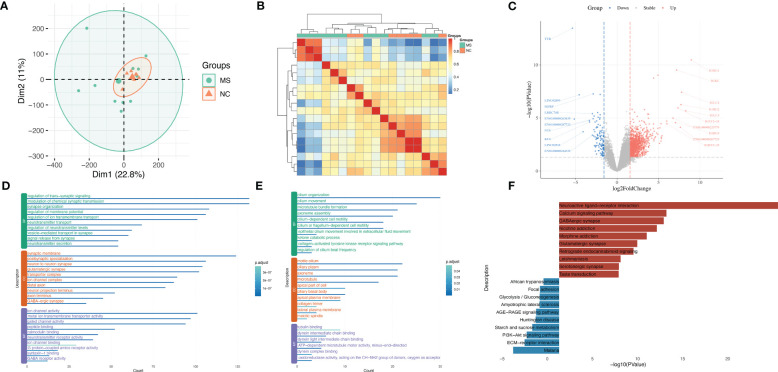
Uncovering gene characteristics during progression of MS. **(A)** PCA of MS samples of GSE214334 datasets including 11 MS white matter tissues and seven non-MS control white matter tissues. **(B)** Correlation heatmap among each MS sample in GSE214334. **(C)** Volcano plot of DEGs between MS and NC. **(D)** GO enrichment analysis of upregulated genes in MS compared with NC. **(E)** GO enrichment analysis of downregulated genes in MS compared with NC. **(F)** KEGG enrichment analysis of upregulated and downregulated genes in MS compared with NC. MS, multiple sclerosis; PCA, principal component analysis; DEGs, differentially expressed genes; GO, gene ontology; KEGG, Kyoto Encyclopedia of Genes and Genomes.

In order to identify molecular mechanisms and gene signatures between MS and NSCLC, first, the gene variation during the progression of MS was evaluated. A total of gene expression profiles of 11 MS white matter tissues and seven non-MS control white matter tissues were analyzed. PCA of these samples showed different gene expression patterns of MS tissue and relatively concentrated patterns of NC tissue (**Figure 2A**), which was possibly a result of different subtypes of MS. Although there were differences between MS subtypes, the correlation of samples suggested the potential correlation among MS (**Figure 2B**).

After calculating the standard deviation expression of genes (**Figure S1A**) and dividing samples into two groups, we obtained DEGs between MS and normal control (**Figure 2C**). IGHG1, IGKC, IGLC2, and IGHG2 were significantly upregulated in MS, while MFRP, NTS, KC6, and TTR were significantly downregulated in MS. A portion of important genes was exhibited by heatmap (**Figure S1B**). GO analysis showed that upregulated genes in MS were enriched in the regulation of trans-synaptic signaling, synaptic membrane, and ion channel activity (**Figure 2D**), while downregulated genes in MS were enriched in cilium organization, motile cilium, and tubulin binding (**Figure 2E**). KEGG analysis showed that upregulated genes in MS were enriched in the Neuroactive ligand–receptor interaction and Calcium signaling pathway, while downregulated genes in MS were enriched in Malaria and ECM–receptor interaction (**Figure 2F**).

### Identification of the unique gene signatures of NSCLC

**Figure 3:**
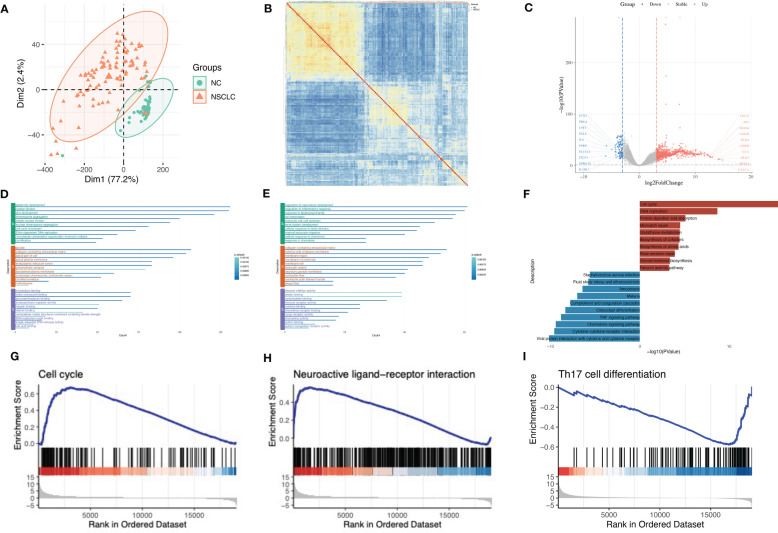
Identification of the unique gene signatures of NSCLC. **(A)** PCA of NSCLC samples of the GSE19188 dataset including 191 NSCLC and 65 adjacent normal lung tissue samples. **(B)** Correlation heatmap among NSCLC samples in GSE19188. **(C)** Volcano plot of DEGs between NSCLC and NC. **(D)** GO enrichment analysis of upregulated genes in NSCLC compared with NC. **(E)** GO enrichment analysis of downregulated genes in NSCLC compared with NC. **(F)** KEGG enrichment analysis of upregulated and downregulated genes in NSCLC compared with NC. **(G)** GSEA results between NSCLC and NC in Cell cycle. **(H)** GSEA results between NSCLC and NC in Neuroactive ligand−receptor interaction. **(I)** GSEA results between NSCLC and NC in Pentose and glucuronate interconversions. NSCLC, non-small cell lung cancer; PCA, principal component analysis; DEGs, differentially expressed genes; NC, normal control; GO, gene ontology; KEGG, Kyoto Encyclopedia of Genes and Genomes; GSEA, Gene Set Enrichment Analysis.

Next, we sought to unveil the unique gene signatures of NSCLC. By integrating 191 NSCLC and 65 adjacent normal lung tissue samples in the GSE19188 dataset and performing PCA, we found that there was an obvious distinction between NSCLC and NC (**Figure 3A**). Correlation analysis also showed homogeneity among NSCLC samples (**Figure 3B**).

After calculating the standard deviation expression of genes (**Figure S2A**) and dividing samples into two groups, we obtained DEGs between NSCLC and NC (**Figure 3C**). CHGA, SST, DLK1, and PCK1 were significantly upregulated in NSCLC, while IL6, IL1RL1, SELE, and S100A12 were significantly downregulated in NSCLC. A portion of important genes is exhibited by heatmap (**Figure S2B**).

GO analysis showed that upregulated genes in NSCLC were enriched in epidermis development, spindle, and microtubule binding (**Figure 3D**), while downregulated genes in NSCLC were enriched in regulation of vasculature development, collagen-containing extracellular matrix, and enzyme inhibitor activity (**Figure 3E**). KEGG analysis showed that upregulated genes in NSCLC were enriched in Cell cycle and DNA replication, while downregulated genes in NSCLC were enriched in viral protein interaction with cytokine and cytokine receptor, Cytokine–cytokine receptor interaction, and Chemokine signaling pathway (**Figure 3F**).

Indeed, GSEA results showed NSCLC was significantly enriched in the Cell cycle (normalized enrichment score (NES) = 1.68, q value < 0.001) and Neuroactive ligand−receptor interaction (NES = 1.44, q value < 0.001) and significantly not enriched in Th17 cell differentiation (NES = −2.19, q value < 0.001) (**Figures 3G–I**).

### Identification of shared gene signatures between MS and NSCLC

**Figure 4:**
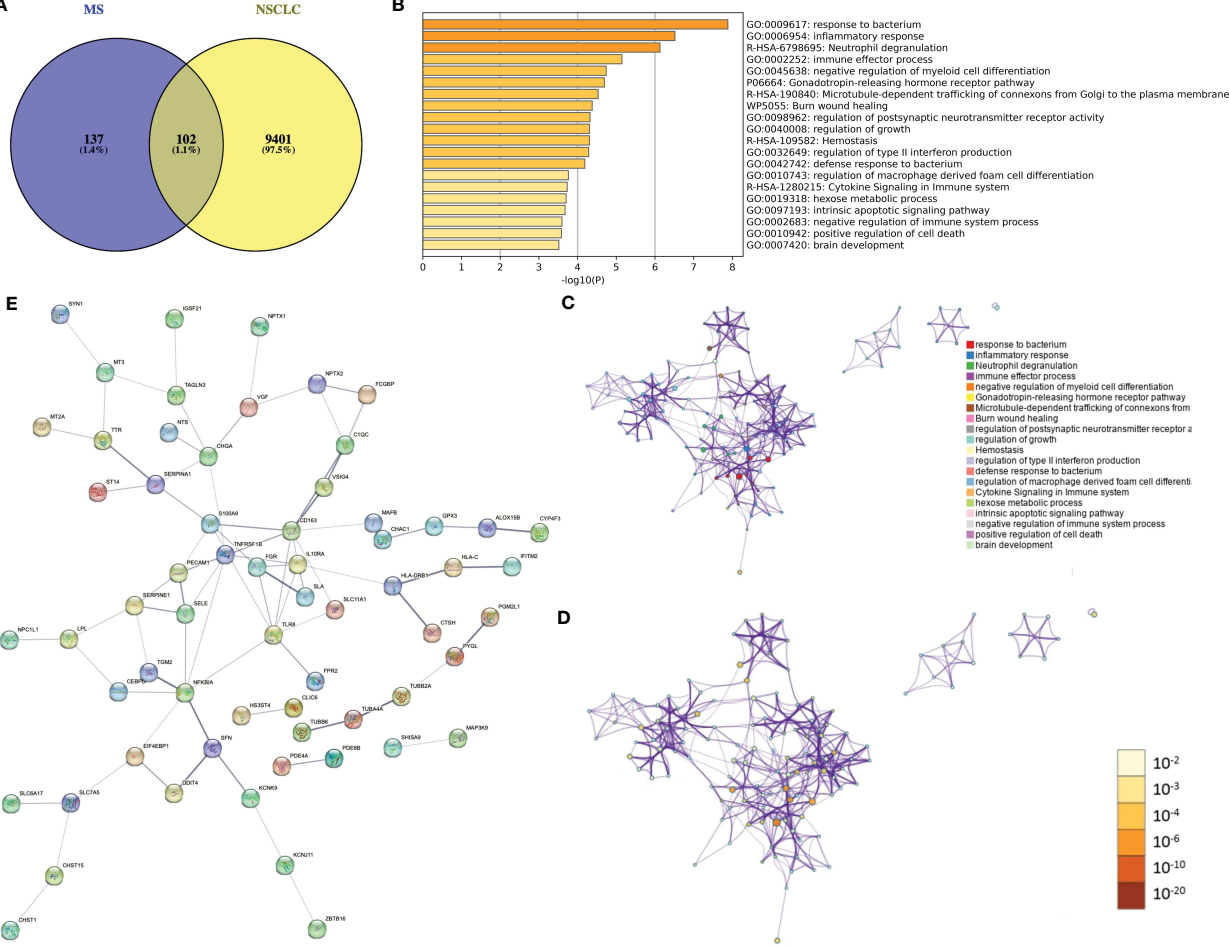
Identification of shared gene signatures between MS and NSCLC. **(A)** Venn plot of shared DEGs between MS and NSCLC. **(B)** Enrichment analysis of shared gene signatures. **(C)** Co-expression network of diverse DEGs. **(D)** Co-expression network of diverse DEGs exhibited by p-value. **(E)** PPI network of shared DEGs between MS and NSCLC. MS, multiple sclerosis; NSCLC, non-small cell lung cancer; DEGs, differentially expressed genes; PPI, protein–protein interaction.

A total of 239 DEGs between MS and NC (false discovery rate (FDR) < 0.05) and 9,503 DEGs between NSCLC and NC (FDR < 0.05) were identified. Using the Venn tool, we finally obtained 102 shared genes, including PDE4A, TTR, NPTX2, IGHG1, ALOX15B, CD163, MT3, and IL1RL1 (**Figure 4A**). Enrichment analysis showed the shared genes enriched response to the bacterium, inflammatory response, and immune effector process (**Figure 4B**). Co-expression network showed potential correlations among these shared genes (**Figures 4C, D**). PPI network analysis showed a shared regulatory network between MS and NSCLC (**Figure 4E**). A portion of node genes, including CD163, IL10RA, TLR8, and NFKBIA, were identified, implying the potential shared mechanisms between MS and NSCLC, such as the formation of the local immune environment and cell motility.

### Further analysis of shared molecular mechanisms between MS and NSCLC

**Figure 5:**
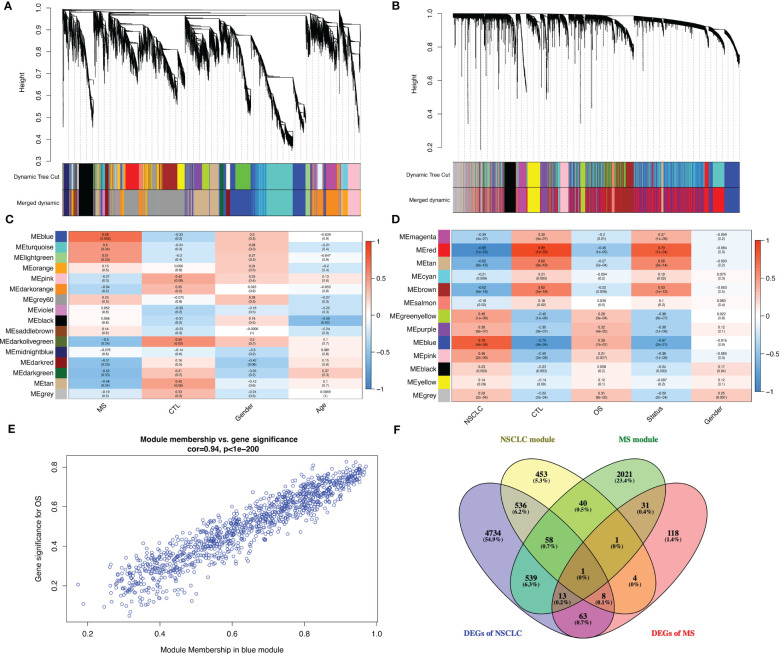
Further analysis of shared molecular mechanisms between MS and NSCLC. Cluster dendrogram of co-expressed genes in MS **(A)** and NSCLC **(B)**. Heatmap of module–trait relationships in MS **(C)** and NSCLC **(D)**. Scatter plot of gene significance for OS *vs.* module member in blue module **(E)**. **(F)** Venn plot of blue module in MS, blue module in NSCLC, DEGs in MS, and DEGs in NSCLC. MS, multiple sclerosis; NSCLC, non-small cell lung cancer; OS, overall survival; DEGs, differentially expressed genes.

We used WGCNA with adequate soft power for the further analysis of shared molecular mechanisms between MS and NSCLC (**Figures S3A, D**). MS patients were divided into relapse–remitting MS, primary progressive MS, and secondary progressive MS groups. By performing the analysis on GSE214334, we obtained 16 modules of genes closely related to the occurrence of MS compared to normal white matter (**Figure 5A**). WGCNA of GSE19188 showed 13 modules of genes closely related to the occurrence of NSCLC compared to normal lung tissue (**Figure 5B**). These modules showed respective associations with other modules (**Figures S3B, E**) and formed specific gene networks (**Figures S3C, F**). The blue module in GSE214334 showed the highest correlation with MS occurrence (R = 0.68, p = 0.002), while the blue module in GSE19188 showed the highest correlation with NSCLC occurrence (R = 0.79, p < 0.001) (**Figures 5C, D**). Moreover, the blue module in GSE214334 showed a correlation with the gender of MS patients (R = 0.3, p = 0.2), while the gray module in GSE19188 showed a significant correlation with the gender of NSCLC patients (R = 0.25, p = 0.001).

There was a high (R = 0.94, p < 0.001) significant correlation between gene significance for overall survival (OS) module members in the blue module (**Figure 5E**). Venn plot shows the blue module in MS, blue module in NSCLC, DEGs in MS, and DEGs in NSCLC. Particularly, in order to improve accuracy, we introduce LUSC and GTEx lung tissue RNA-seq data and successfully identified the DEGs (log2FC > 1, q value < 0.05), with a total of 5,964 genes. Finally, we obtained one shared gene, PDE4A, which might be the most important shared gene during the progression of these two diseases (**Figure 5F**).

### The expression of PDE4A and its impact on the survival of NSCLC patients

**Figure 6:**
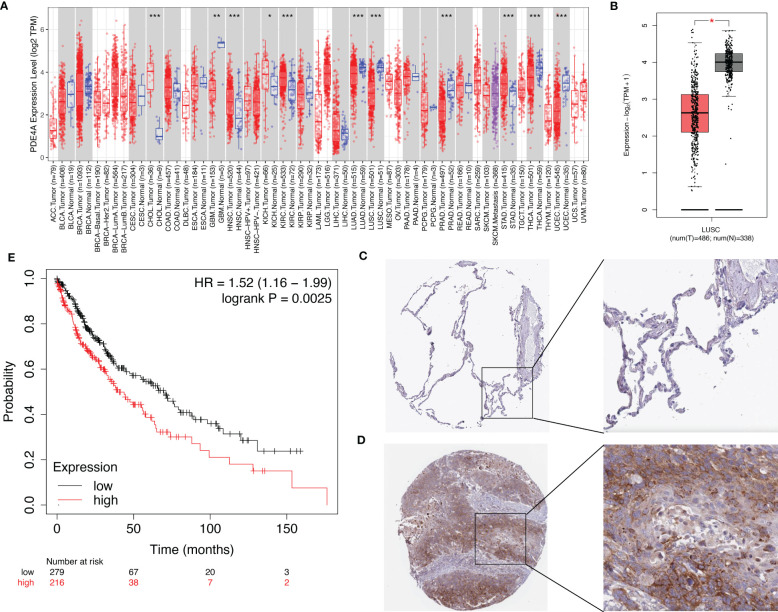
The expression of PDE4A and its impact on survival of NSCLC patients. **(A)** The expression of PDE4A on various cancers and normal tissues. **(B)** The expression of PDE4A on LUSC. **(C)** The protein level of PDE4A on normal lung tissue test by IHC. **(D)** The protein level of PDE4A on NSCLC tumor tissue test by IHC. **(E)** The impact of PDE4A expression on survival of NSCLC patients. NSCLC, non-small cell lung cancer; LUSC, lung squamous cell carcinoma; IHC, immunohistochemistry. *p<0.05 , **p<0.01, ***p<0.001.

Next, we speculated whether PDE4A had different expression patterns in normal and tumor tissues. Indeed, it seemed that PDE4A was differentially expressed in diverse cancer types (**Figure 6A**). For example, PDE4A was expressed significantly higher in stomach adenocarcinoma rather than in normal stomach tissue, and it was expressed significantly higher in cholangiocarcinoma rather than in normal biliary tract tissue, which suggested a crucial role of PDE4A in cancer progression. Moreover, PDE4A was expressed significantly lower in NSCLC tissue compared with normal lung tissue (**Figure 6B**). However, the protein level of PDE4A was opposite from the RNA level, suggesting potential post-translational regulation (**Figures 6C, D**). Survival analysis showed NSCLC patients with higher PDE4A expression levels had worse prognoses, indicating the pro-tumor role of PDE4A (**Figure 6E**).

### The influence of PDE4A expression on molecular mechanisms during the progression of NSCLC

**Figure 7:**
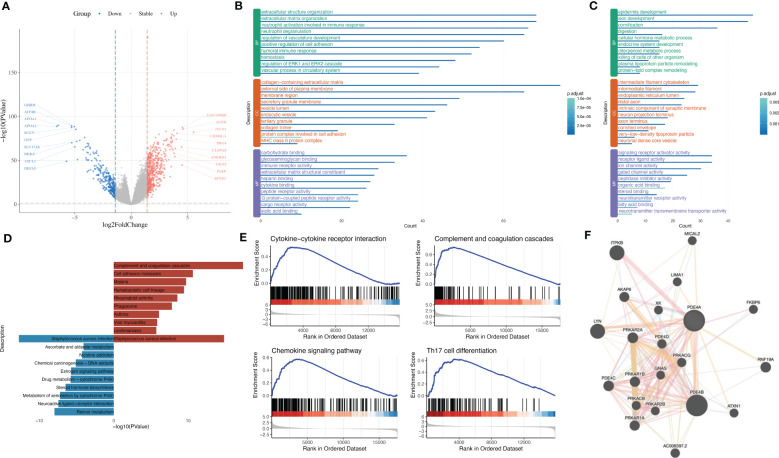
The influence of PDE4A expression on molecular mechanisms during the progression of NSCLC. **(A)** Volcano plot shows DEGs between PDE4A high-expression group and PDE4A low-expression group. **(B)** GO enrichment analysis of significantly upregulated genes in PDE4A high-expression group. **(C)** GO enrichment analysis of significantly downregulated genes in PDE4A high-expression group. **(D)** KEGG enrichment analysis of upregulated and downregulated genes in PDE4A high-expression group. **(E)** GSEA results between PDE4A high-expression group and PDE4A low-expression group. **(F)** PPI network of PDE4A. NSCLC, non-small cell lung cancer; DEGs, differentially expressed genes; GO, gene ontology; KEGG, Kyoto Encyclopedia of Genes and Genomes; GSEA, Gene Set Enrichment Analysis; PPI, protein–protein interaction.

In order to figure out how PDE4A affects the progression of NSCLC, we divided NSCLC patients into two groups: the PDE4A high-expression group and the PDE4A low-expression group. The volcano plot shows that there were different expressed patterns between the PDE4A high-expression group and the PDE4A low-expression group (**Figure 7A**). The PDE4A high-expression group expressed significantly higher levels of AGER, ITLN1, GKN2, and PAEP, whereas it expressed significantly lower levels of GHRH, ATP4B, APOA1, and APOA2.

GO analysis showed that upregulated genes in the PDE4A high-expression group were enriched in extracellular structure organization, collagen-containing extracellular matrix, and carbohydrate binding (**Figure 7B**), while downregulated genes in the PDE4A high-expression group were enriched in epidermis development, intermediate filament cytoskeleton, and signaling receptor activator activity (**Figure 7C**). KEGG analysis showed that upregulated genes in the PDE4A high-expression group were enriched in complement and coagulation cascades and malaria, while downregulated genes in NSCLC were enriched in retinol metabolism and drug metabolism-cytochrome P450 (**Figure 7D**).

GSEA results showed the PDE4A high-expression group was significantly enriched in complement and coagulation cascades (NES = 2.07, q value < 0.001), chemokine signaling pathway (NES = 1.90, q value < 0.001), Th17 cell differentiation (NES = 2.19, q value < 0.001), and cytokine–cytokine receptor interaction (NES = 2.11, q value < 0.001) (**Figure 7E**). The PPI network of PDE4A showed that PDE4A, PDE4D, and PDE4B might integrally work together to influence the progression of NSCLC (**Figure 7F**).

### The association between PDE4A and the local immune environment in NSCLC

**Figure 8:**
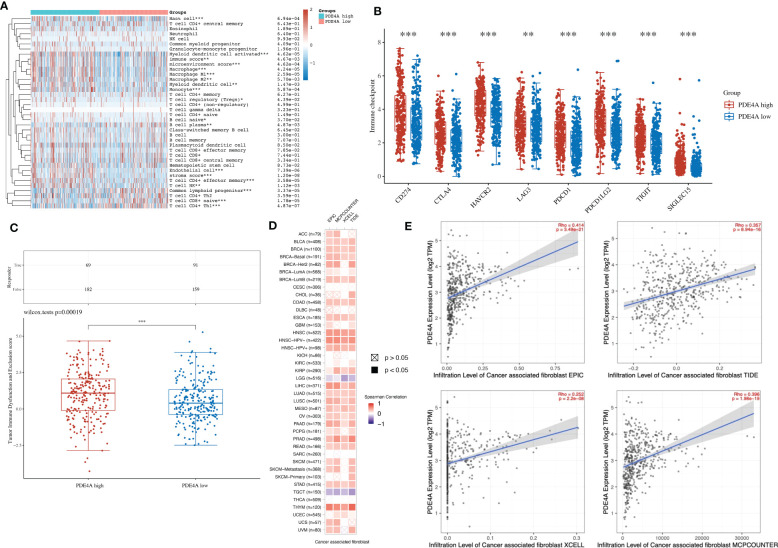
The association between PDE4A and local immune environment in NSCLC. **(A)** The association between PDE4A expression and local immune environment in NSCLC using the XCELL method. **(B)** The association between PDE4A expression and immune checkpoint in NSCLC. **(C)** The association between PDE4A expression and immune checkpoint blockade in NSCLC. **(D)** The correlation heatmap between PDE4A expression and local cancer-associated fibroblast in NSCLC. **(E)** The scatter plot of correlation between PDE4A expression and local cancer-associated fibroblast in NSCLC using different methods. NSCLC, non-small cell lung cancer. as with immune checkpoint. *p<0.05 , **p<0.01, ***p<0.001.

Considering that PDE4A could influence the immune process according to the above analysis, we next tried to elucidate the association between PDE4A and the local immune environment in NSCLC. PDE4A expression showed a significantly high association with the local immune environment in NSCLC (**Figure 8A**), as with immune checkpoint (**Figure 8B**) and immune checkpoint blockade (**Figure 8C**).

Interestingly, PDE4A expression showed a significant positive correlation with cancer-associated fibroblast in most cancer types (**Figure 8D**). As for NSCLC, PDE4A expression also showed a significant positive correlation with cancer-associated fibroblast in all methods: EPIC (R = 0.414, p < 0.001), MCPCOUNTER (R = 0.396, p < 0.001), XCELL (R = 0.252, p < 0.001), and TIDE (R = 0.357, p < 0.001) (**Figure 8E**).

### Diversified treatment response of NSCLC with different PDE4A expression levels

**Figure 9:**
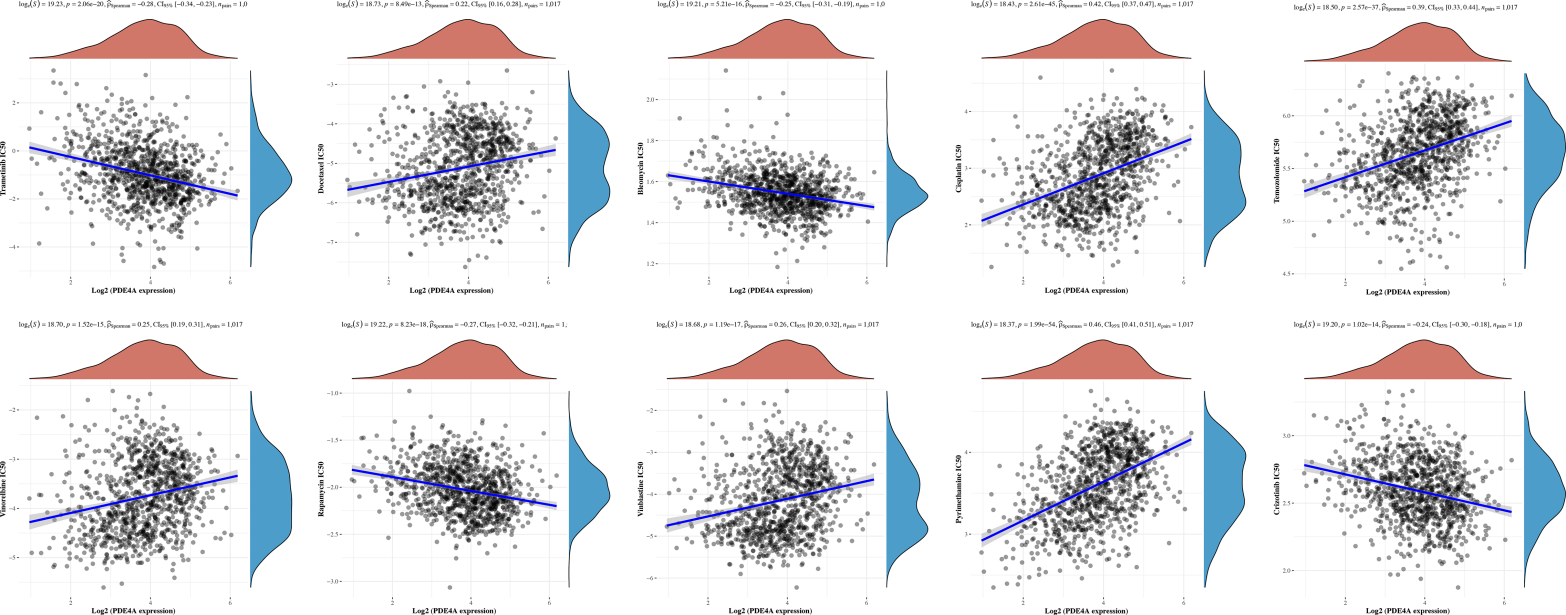
Diversified treatment response of NSCLC with different PDE4A expression. NSCLC, non-small cell lung cancer.

Finally, we sought to figure out whether PDE4A expression would affect the treatment response of different NSCLC drugs. According to **Figure 9**, PDE4A expression has specific impacts on the treatment response of trametinib (R^2^ = −0.28, p < 0.001), docetaxel (R^2^ = 0.22, p < 0.001), bleomycin (R^2^ = −0.25, p < 0.001), cisplatin (R^2^ = 0.42, p < 0.001), temozolomide (R^2^ = 0.39, p < 0.001), vinorelbine (R^2^ = 0.25, p < 0.001), rapamycin (R^2^ = −0.27, p < 0.001), vinblastine (R^2^ = 0.26, p < 0.001), pyrimethamine (R^2^ = −0.28, p < 0.001), and crizotinib (R^2^ = −0.24, p < 0.001) during treatment of NSCLC.

## Discussion

With the development of society, chronic diseases and comorbidities (including complex diseases) have become the main health problems and causes of death. MS, the most common demyelinating disease of the central nervous system, has caused pain and inconvenience to more than 2 million people worldwide. Although multiple sclerosis itself does not cause death, a number of complications can seriously affect the quality of life and mortality of patients. The prospective cohort study by Grytten et al. found that MS patients had a higher risk of cancer than the population control group, with a 66% increased risk of respiratory system tumors at 65 years of age (1). NSCLC is the most prevalent type of lung cancer, accounting for 85% of all lung cancer cases (7). Most patients are diagnosed at an advanced stage, resulting in poor prognoses. The association between MS and NSCLC has been demonstrated in previous clinical cohorts, and earlier studies have explored genomic profiles associated with MS and NSCLC (8, 9). However, the molecular mechanisms underlying this association have not been comprehensively studied.

It has been reported that immune dysregulation, both locally and systemically, plays a pivotal role in the development of MS (10). Similarly, there is compelling evidence that the development of NSCLC is closely related to alterations in the tumor immune microenvironment (11). Given these observations, it is intriguing to ask whether the two diseases share similar immune microenvironments during their pathogenesis. To address this question, we conducted a joint analysis to identify characteristic genes involved in the development of both lung cancer and MS. Our investigation resulted in the identification of 102 common genes, including PDE4A, TTR, NPTX2, IGHG1, ALOX15B, CD163, MT3, and IL1RL1. Significantly, this set of common genes includes a large number of immune-related genes, suggesting that the two diseases may share similar alterations in the immune microenvironment during their pathogenesis. Moreover, pathway enrichment analysis supports the involvement of immune responses, immune effector processes, and cytokine signaling pathways in the common pathogenesis of these two diseases.

In order to investigate the potential relationship between MS and NSCLC, we employed WGCNA to identify gene modules associated with the development of these two diseases. We then conducted a joint analysis of the modular genes most closely related to the development of both diseases, identifying overlapping genes that are considered shared genes and may be related to the pathogenesis of both MS and NSCLC. Additionally, we examined the biological processes and signaling pathways in which these shared genes are involved. Intriguingly, enrichment analysis revealed that these shared genes were enriched in responses to bacteria, inflammatory reactions, and immune effects, which are known to be associated with the pathogenesis of both MS and NSCLC (10, 12, 13). Further analysis of a subset of nodal genes, including CD163, IL10RA, TLR8, and NFKBIA, indicated potential shared mechanisms between MS and NSCLC, such as the formation of local immune environments and cell movement. To enhance our findings, we integrated TCGA LUSC and GTEx lung tissue RNA-seq data and ultimately identified only one common gene, PDE4A, which may be the most crucial shared gene in the progression of both diseases. Furthermore, we used single-cell sequencing data to evaluate the local immune microenvironment of MS and NSCLC and observed some similar immune cell components in the immune microenvironments of both diseases, such as T cells and fibroblasts. Interestingly, we also observed that PDE4A expression showed a significant positive correlation with cancer-associated fibroblast in most cancer types. As for NSCLC, PDE4A expression also showed a significant positive correlation with cancer-associated fibroblast. Based on these findings, we can cautiously hypothesize that the development of both diseases may involve some potential similar changes in the immune microenvironment, which may contribute to the further malignant progression of lung cancer.

The PDE4 family of phosphodiesterases has been identified as the most diverse among all PDE families, with enzymes that are widely distributed in various tissues and present in all major organs, including the brain. Furthermore, PDE4 is abundant in immune and inflammatory cells in lung diseases such as asthma (14). The PDE4 family is mainly composed of four gene products, PDE4A, PDE4B, PDE4C, and PDE4D, as well as several N-terminal splice variants that differ in their tissue and cell expression patterns (15). The hallmark of this group of enzymes is their high affinity for cAMP and insensitivity to cGMP and calmodulin (16). Specifically, PDE4A is capable of hydrolyzing the second messenger cyclic adenosine monophosphate (cAMP) with a micromolar Km value and acts as a regulator and mediator of many cell-to-cell signaling responses, thereby playing a critical role in numerous important physiological processes by modulating the cellular concentration of cAMP (17). In recent years, the PDE4 family has been the subject of extensive research due to its significance as a major therapeutic target for intervention in various inflammatory diseases, such as asthma, chronic obstructive pulmonary disease (COPD), and rheumatoid arthritis (RA). This is primarily because cAMP-specific PDEs, particularly PDE4, are the main subtypes of PDE that regulate the activity of inflammatory cells. Consequently, several PDE4 selective inhibitors have been developed for the treatment of inflammatory diseases, including Zoryve emulsion for psoriasis and roflumilast for asthma.

There are some existing studies on PDE4A in MS and lung cancer. MS is characterized by chronic neuroinflammation, demyelination, and destruction of oligodendrocytes, axons, and neurons. Pro-inflammatory cytokines, including interferon-gamma (IFN-gamma), tumor necrosis factor-α (TNF-α), interleukin-6 (IL-6), interleukin-12 (IL-12), and interleukin-23 (IL-23), are crucial in the pathogenesis of MS (18, 19). Evidence suggests that cAMP is a crucial participant in regulating the production of pro-inflammatory cytokines (20). Notably, cAMP analogs have been shown to have anti-inflammatory and anti-apoptotic effects. Previous studies have suggested that Dibutyryl-cAMP recruits endogenous neural stem cells and promotes their differentiation, thereby facilitating myelin lipid repair in MS (21). Given the critical role of PDE4A in cAMP hydrolysis, it is reasonable to hypothesize that this protein contributes to the pathogenesis of MS. Previous studies have also highlighted the importance of PDE4A in various tumors (22). In particular, PDE4A expression is upregulated in various lung cancer cell lines, and its expression can induce epithelial–mesenchymal transition in type 1 alveolar epithelial cells after stimulation with transforming growth factor-β2 (TGF-β2). E-cadherin loss, which is a hallmark of metastasis, is significantly associated with the upregulation of PDE4A mRNA and protein expression after stimulation with transforming growth factor-β1 (TGF-β1) (23). Moreover, hypoxia-inducible factor (HIF) has been linked to PDE4A expression in a subset of lung cancer cell lines during hypoxia, and PDE4A knockdown has been shown to reduce the secretion of vascular endothelial growth factor (VEGF) and has anti-tumor effects in lung cancer xenografts (24). Thus, it is clear that PDE4A is a promising therapeutic target in MS and lung cancer and warrants further investigation.

In our investigation, we employed a systemic biology analysis method to identify the genes with the highest correlation in the comorbidity of MS and NSCLC. Among the genes analyzed, PDE4A exhibited the highest correlation. Notably, patients with high PDE4A expression showed poor prognoses, suggesting that this gene may be associated with increased incidence and mortality of NSCLC in MS patients. Our investigation further revealed the regulatory effects of PDE4A on other immune cells and its association with immune checkpoints, indicating its significant role in regulating the human immune response in NSCLC. Additionally, our findings demonstrated that PDE4A was closely associated with the sensitivity of multiple NSCLC therapeutic drugs. Future research targeting PDE4A therapy may improve the incidence and mortality of NSCLC in MS patients.

However, it is important to acknowledge the limitations of our study. Although our findings suggest a potential molecular mechanism linking MS and NSCLC, our results are based solely on data analysis and lack experimental validation, which is not quite a comprehensive analysis. Therefore, further investigation is required to confirm our hypothesis and establish a causal relationship. Nonetheless, our study has contributed novel insights into the comorbidity of MS and NSCLC and has identified PDE4A as a promising therapeutic target and immune-related biomarker for these patients.

## Data availability statement

The original contributions presented in the study are included in the article/**Supplementary Material**. Further inquiries can be directed to the corresponding authors.

## Author contributions

JY and BN designed the study. JY, XH, YW, WL, and MZ performed the data analysis. JY drafted the manuscript. AZ and BN revised the manuscript. All authors contributed to the article and approved the submitted version.

## Supplementary material

Supplementary Figure 1 Gene expression distribution of MS. **(A)** Top 100 standard deviation genes of MS. **(B)** Heatmap of representative DEGs between MS and NC.

Supplementary Figure 2 Gene expression distribution of NSCLC. **(A)** Top 100 standard deviation genes of NSCLC. **(B)** Heatmap of representative DEGs between NSCLC and NC.

Supplementary Figure 3 WGCNA of MS and NSCLC. Soft power distribution of MS **(A)** and NSCLC **(D)**. Correction heatmap among gene modules of MS **(B)** and NSCLC **(E)**. Visualization of the gene network of MS **(C)** and NSCLC **(F)** using heatmap.

## References

[1] Grytten N Myhr KM Celius EG Benjaminsen E Kampman M Midgard R. Risk of cancer among multiple sclerosis patients, siblings, and population controls: a prospective cohort study. Mult Scler (2020) 26:1569–80. doi: 10.1177/1352458519877244 31573834

[2] Lunde H Assmus J Myhr KM Bo L Grytten N. Survival and cause of death in multiple sclerosis: a 60-year longitudinal population study. J Neurol Neurosurg Psychiatry (2017) 88:621–25. doi: 10.1136/jnnp-2016-315238 PMC5537547 28365589

[3] Tian DC Zhang C Yuan M Yang X Gu H Li Z. Incidence of multiple sclerosis in China: a nationwide hospital-based study. Lancet Reg Health West Pac (2020) 1:100010. doi: 10.1016/j.lanwpc.2020.100010 34327341 PMC8315658

[4] Garcia CR Jayswal R Adams V Anthony LB Villano JL. Multiple sclerosis outcomes after cancer immunotherapy. Clin Transl Oncol (2019) 21:1336–42. doi: 10.1007/s12094-019-02060-8 PMC6702101 30788836

[5] Oliveira M de Brito MH Simabukuro MM. Central nervous system demyelination associated with immune checkpoint inhibitors: review of the literature. Front Neurol (2020) 11:538695. doi: 10.3389/fneur.2020.538695 33362680 PMC7759512

[6] Ward M Goldman MD. Epidemiology and pathophysiology of multiple sclerosis. Continuum (Minneap Minn) (2022) 28:988–1005. doi: 10.1212/CON.0000000000001136 35938654

[7] Kastner J Hossain R White CS. Epidemiology of lung cancer. Semin Roentgenol (2020) 55:23–40. doi: 10.1053/j.ro.2019.10.003 31964477

[8] Nikolaos AP Sergio EB Adam S Parisa S Chris C Garrett W. Multiple sclerosis genomic map implicates peripheral immune cells and microglia in susceptibility. Science (2019) 365(6460):eaav7188. doi: 10.1126/science.aav7188 31604244 PMC7241648

[9] Kim TM Yim SH Lee JS Kwon MS Ryu JW Kang HM. Genome-wide screening of genomic alterations and their clinicopathologic implications in non-small cell lung cancers. Clin Cancer Res (2005) 11:8235–42. doi: 10.1158/1078-0432.CCR-05-1157 16322280

[10] Rodriguez MS Farez MF Quintana FJ. The immune response in multiple sclerosis. Annu Rev Pathol (2022) 17:121–39. doi: 10.1146/annurev-pathol-052920-040318 34606377

[11] Genova C Dellepiane C Carrega P Sommariva S Ferlazzo G Pronzato P. Therapeutic implications of tumor microenvironment in lung cancer: focus on immune checkpoint blockade. Front Immunol (2021) 12:799455. doi: 10.3389/fimmu.2021.799455 35069581 PMC8777268

[12] Hoftberger R Lassmann H. Inflammatory demyelinating diseases of the central nervous system. Handb Clin Neurol (2017) 145:263–83. doi: 10.1016/B978-0-12-802395-2.00019-5 PMC7149979 28987175

[13] Sautes-Fridman C Cherfils-Vicini J Damotte D Fisson S Fridman WH Cremer I. Tumor microenvironment is multifaceted. Cancer Metastasis Rev (2011) 30:13–25. doi: 10.1007/s10555-011-9279-y 21271351

[14] Franciosi LG Diamant Z Banner KH Zuiker R Morelli N Kamerling IM. Efficacy and safety of RPL554, a dual PDE3 and PDE4 inhibitor, in healthy volunteers and in patients with asthma or chronic obstructive pulmonary disease: findings from four clinical trials. Lancet Respir Med (2013) 1:714–27. doi: 10.1016/S2213-2600(13)70187-5 24429275

[15] BinMahfouz H Borthakur B Yan D George T Giembycz MA Newton R. Superiority of combined phosphodiesterase PDE3/PDE4 inhibition over PDE4 inhibition alone on glucocorticoid- and long-acting beta2-adrenoceptor agonist-induced gene expression in human airway epithelial cells. Mol Pharmacol (2015) 87:64–76. doi: 10.1124/mol.114.093393 25324049

[16] Suvarna NU O'Donnell JM. Hydrolysis of n-methyl-D-aspartate receptor-stimulated cAMP and cGMP by PDE4 and PDE2 phosphodiesterases in primary neuronal cultures of rat cerebral cortex and hippocampus. J Pharmacol Exp Ther (2002) 302:249–56. doi: 10.1124/jpet.302.1.249 12065724

[17] Alex B Maria-Jesus M Sandra O Michele M Shadab A Emanuele A. UniProt: the universal protein knowledgebase in 2023. Nucleic Acids Res (2023) 51:D523–31. doi: 10.1093/nar/gkac1052 PMC9825514 36408920

[18] Trenova AG Slavov GS Draganova-Filipova MN Mateva NG Manova MG Miteva LD. Circulating levels of interleukin-17A, tumor necrosis factor-alpha, interleukin-18, interleukin-10, and cognitive performance of patients with relapsing-remitting multiple sclerosis. Neurol Res (2018) 40:153–59. doi: 10.1080/01616412.2017.1420522 29297273

[19] Deshpande P King IL Segal BM. IL-12 driven upregulation of p-selectin ligand on myelin-specific T cells is a critical step in an animal model of autoimmune demyelination. J Neuroimmunol (2006) 173:35–44. doi: 10.1016/j.jneuroim.2005.11.016 16413063

[20] Bopp T Jonuleit H Schmitt E. Regulatory T cells–the renaissance of the suppressor T cells. Ann Med (2007) 39:322–34. doi: 10.1080/07853890701379700 17701475

[21] Khezri S Javan M Goudarzvand M Semnanian S Baharvand H. Dibutyryl cyclic AMP inhibits the progression of experimental autoimmune encephalomyelitis and potentiates recruitment of endogenous neural stem cells. J Mol Neurosci (2013) 51:298–306. doi: 10.1007/s12031-013-9959-x 23335001

[22] Hsien LS Zervoudakis G Chou J Gurney ME Quesnelle KM. PDE4 subtypes in cancer. Oncogene (2020) 39:3791–802. doi: 10.1038/s41388-020-1258-8 PMC7444459 32203163

[23] Kolosionek E Savai R Ghofrani HA Weissmann N Guenther A Grimminger F. Expression and activity of phosphodiesterase isoforms during epithelial mesenchymal transition: the role of phosphodiesterase 4. Mol Biol Cell (2022) 33:r2. doi: 10.1091/mbc.E09-01-0019_corr PMC9582637 35862500

[24] Pullamsetti SS Banat GA Schmall A Szibor M Pomagruk D Hanze J. Phosphodiesterase-4 promotes proliferation and angiogenesis of lung cancer by crosstalk with HIF. Oncogene (2013) 32:1121–34. doi: 10.1038/onc.2012.136 22525277

