# Supplementary figures and images for: Identification of the shared gene signatures and molecular mechanisms between multiple sclerosis and non-small cell lung cancer
Source: Front Immunol. 2023 May 12;14:1180449. doi: 10.3389/fimmu.2023.1180449 (PMC10213509; doi:10.3389/fimmu.2023.1180449)

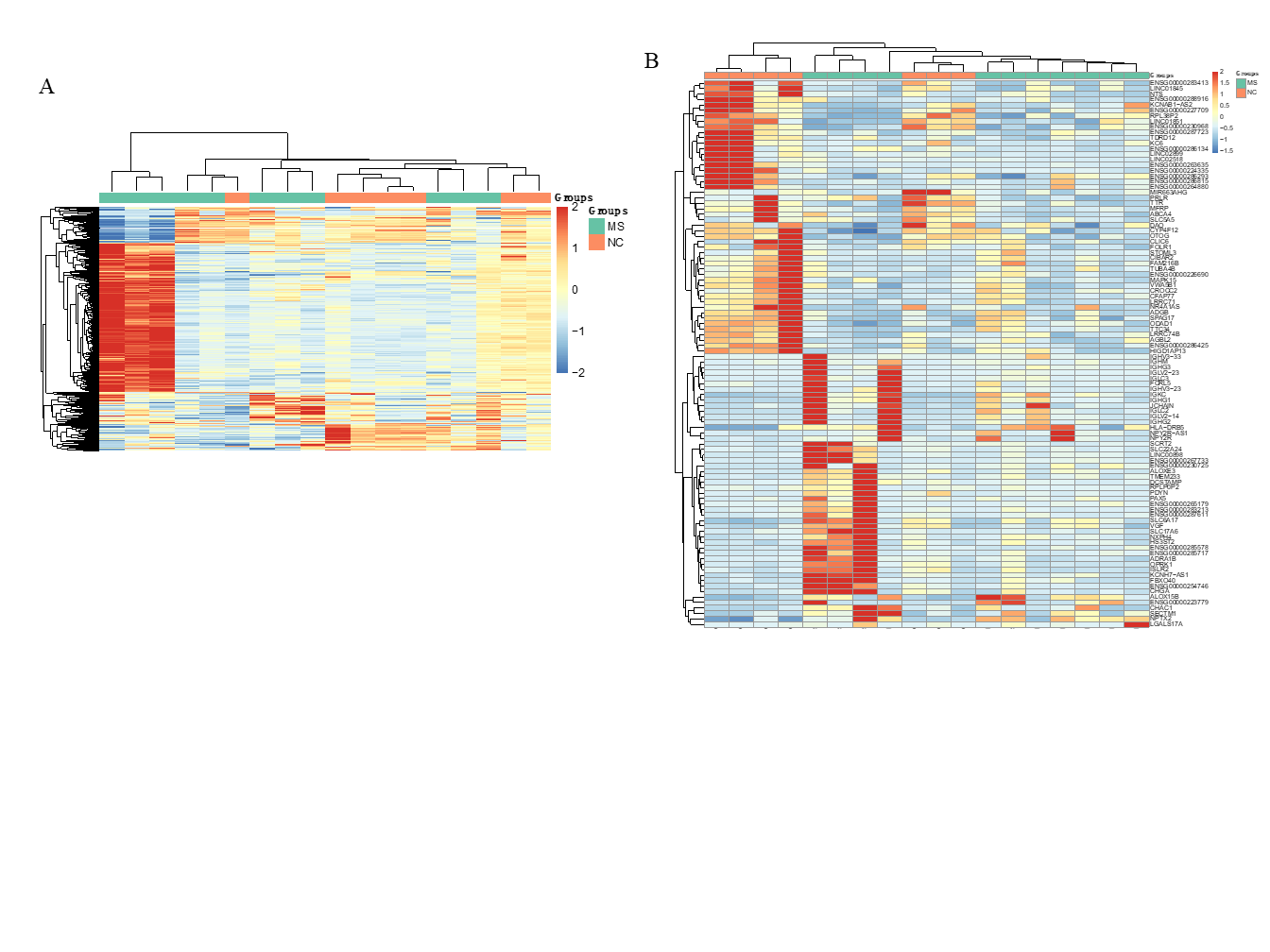

Supplement: Supplementary Figure 1 — Gene expression distribution of MS. (A) Top 100 standard deviation genes of MS. (B) Heatmap of representative DEGs between MS and NC. [file Image_1.png]

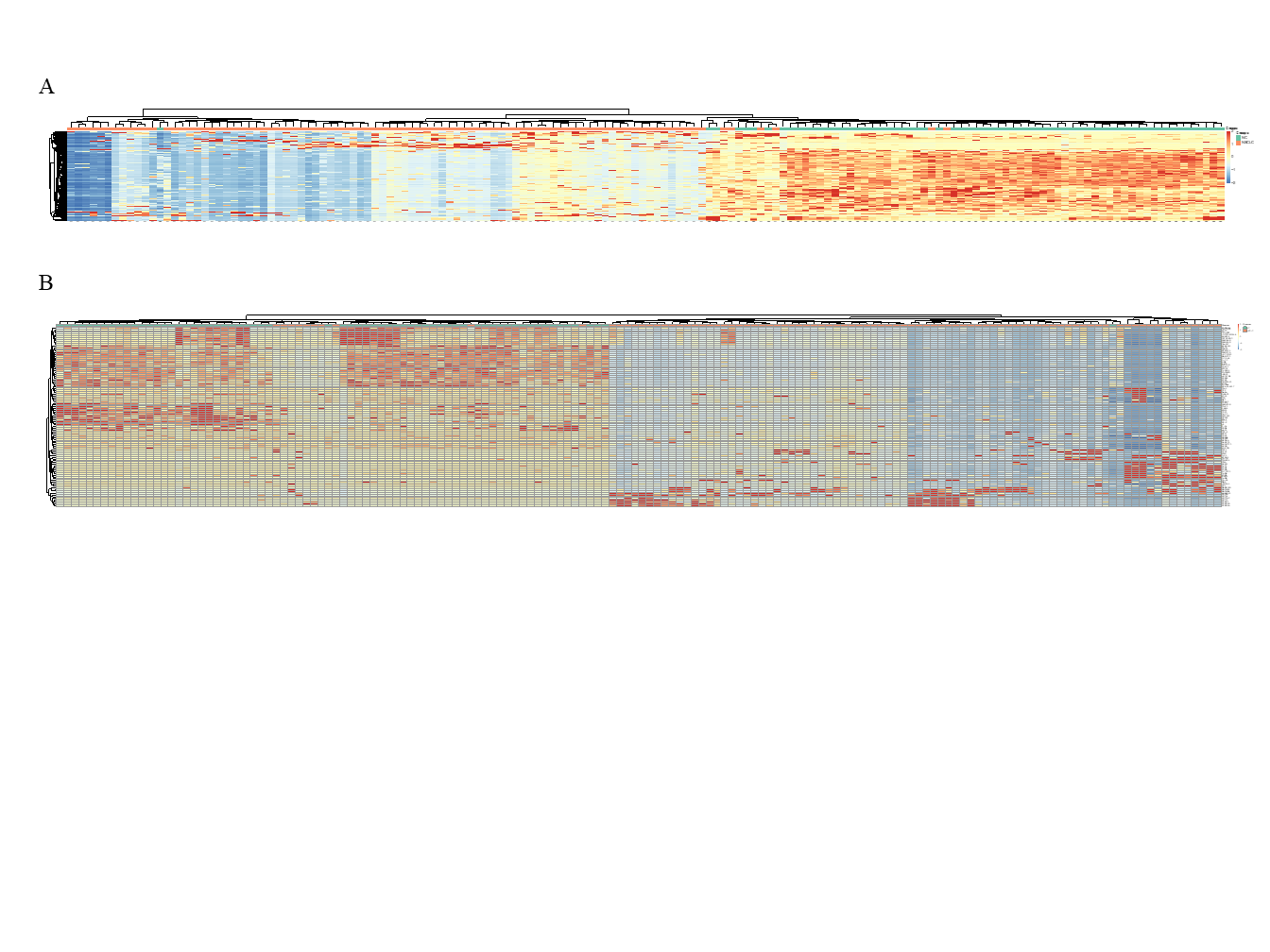

Supplement: Supplementary Figure 2 — Gene expression distribution of NSCLC. (A) Top 100 standard deviation genes of NSCLC. (B) Heatmap of representative DEGs between NSCLC and NC. [file Image_2.png]

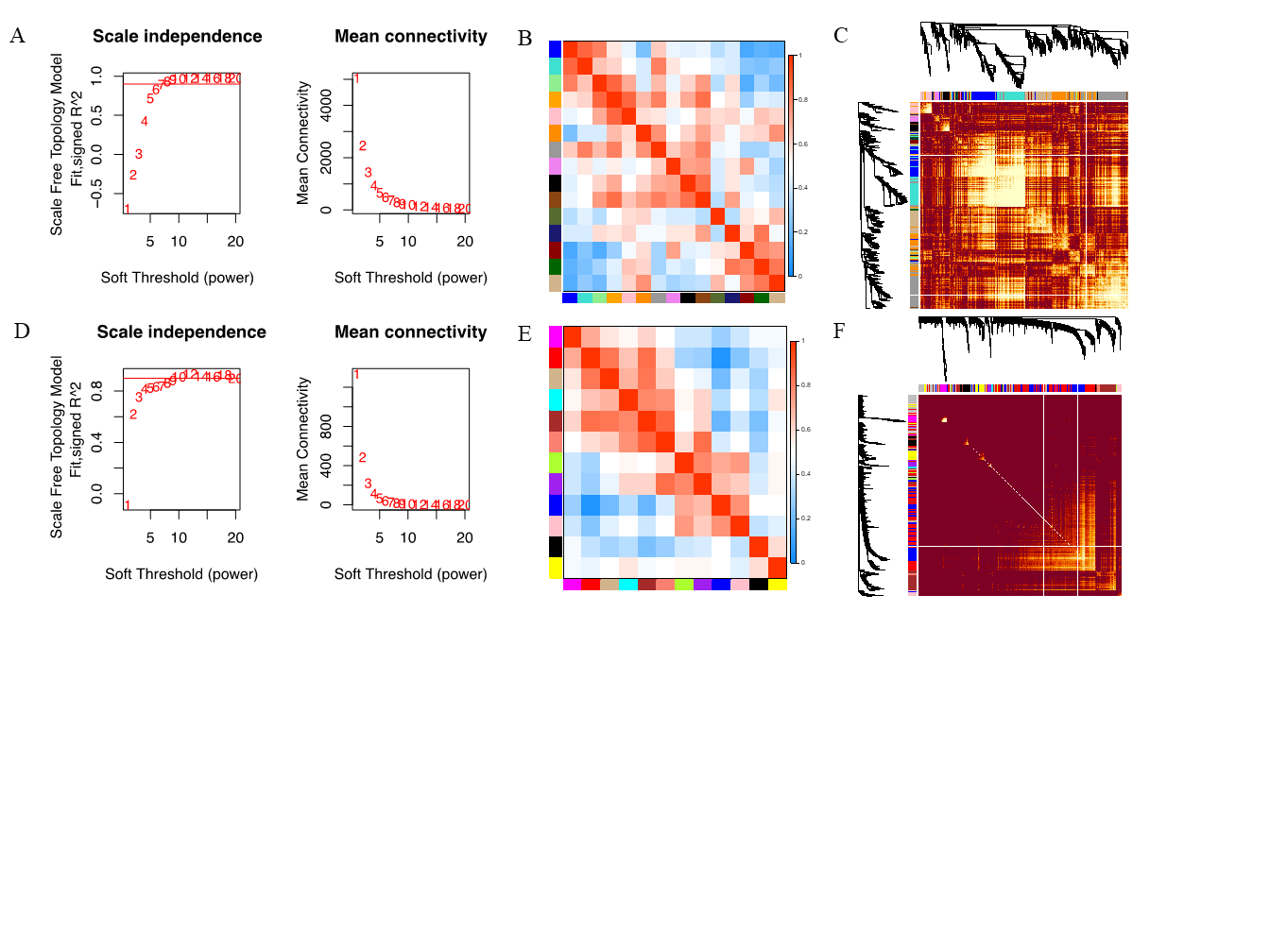

Supplement: Supplementary Figure 3 — WGCNA of MS and NSCLC. Soft power distribution of MS (A) and NSCLC (D). Correction heatmap among gene modules of MS (B) and NSCLC (E). Visualization of the gene network of MS (C) and NSCLC (F) using heatmap. [file Image_3.png]
